# ^18^FDG-PET/CT findings in COVID-19: a single centre retrospective radiological review

**DOI:** 10.1259/bjrcr.20200091

**Published:** 2020-06-26

**Authors:** Pia F. P. Charters, David Little, Jonathan C. L. Rodrigues, Richard N Graham, Stewart L Redman

**Affiliations:** 1 Department of Radiology, Royal United Hospitals, Bath, UK

## Abstract

The severe acute respiratory syndrome coronavirus 2 (SARS-CoV-2), which causes the infectious disease COVID-19, was declared a global pandemic in March 2020. As radiology departments recommence ^18^FDG-PET/CT imaging, it is likely that both asymptomatic and specific symptomatic patients with COVID-19 infection will be imaged, particularly if the disease becomes endemic in the UK. We review the clinical scenarios where ^18^FDG-PET/CT could be performed in COVID-19 positive patients. Our local protocol for safely scanning known COVID-19 positive patients is described, highlighting considerations for other departments.

We present the findings from a series of known COVID-19 positive patients and two further asymptomatic cases evaluated with^18^FDG-PET/CT. Classic, indeterminate, normal and non-COVID-19 manifestations on both the ^18^FDG-PETand low dose CT component are described as an aid for radiologists and nuclear medicine physicians when reporting ^18^FDG PET/CT.

## Introduction

The severe acute respiratory syndrome coronavirus 2 (SARS-CoV-2), which causes the infectious disease COVID-19, was declared a global pandemic in March 2020. Although detection of viral RNA with reverse transcription polymerase chain reaction (RT-PCR) remains the gold-standard for diagnosis, the sensitivity has been reported as low as 60–70%.^1^

No imaging modality is recommended as a first-line investigation to screen or diagnose COVID-19, although imaging may play a role in patient management.^2^

Several cases of COVID-19 have been described incidentally on ^18^FDG-PET/CT, although there is little documented about scanning known positive cases. As radiology departments recommence ^18^FDG-PET/CT imaging, it is likely that asymptomatic patients with undiagnosed COVID-19 infection will be imaged, particularly if COVID-19 becomes endemic in the UK.^3^ The PET/CT reporter will need to be able to describe the COVID-19-related findings on these scans.

Many patients will have symptoms compatible with COVID-19 but be managed in the community and not necessarily receive a formal RT-PCR positive diagnosis. Of those that are swab positive, the clinical course can vary and prolonged illness may ensue due to the systemic inflammatory response elicited by the virus. The radiological sequelae of COVID-19 may persist for a long time regardless of the clinical course or whether the patient is infective.^4,5^ Such post-inflammatory change may cause diagnostic challenge and during this period of uncertainty patients may require ^18^FDG-PET/CT scans as part of their active management. We review the clinical scenarios where ^18^FDG-PET/CT could be performed in COVID-19 positive patients and highlight considerations to enable a safe process for scanning known COVID-19 positive patients in these scenarios.

We present the findings from a series of 6 proven or suspicious COVID-19 patients evaluated with ^18^FDG-PET/CT at our (Royal United Hospitals, Bath) institution, highlighting the spectrum of imaging findings.

Classic, indeterminate, normal and non-COVID-19 manifestations on both the ^18^FDG-PET and low dose CT component are described as an aid for radiologists and nuclear medicine physicians when reporting ^18^FDG PET/CT.

### Indications of PET-CT in COVID-19

When referred a COVID-19 positive patient, consideration is given to the clinical indication.

Our local approach has been adapted from the British Nuclear Medicine Society (BNMS) decision-making tool, which aids Nuclear Medicine reporters when vetting studies for symptomatic or positive COVID-19 patients^3,6^:

- In non-urgent cases including non-aggressive cancer types or follow-up scans, a delay of up to 4–6 weeks is considered to ensure that the patient is well enough to undergo the scan and unlikely to be infectious to staff in the department.
- When referred more urgent cases such as new cancer, we aim to delay by 1–2 weeks to balance the risks of COVID-19 with the need to progress disease management. These appointments must be discussed with a clinician beforehand if considering cancelling/rebooking.
- Some complex inflammatory/sepsis patients may require an urgent ^18^FDG-PET/CT scan whilst actively infected with COVID-19.

### Scanning procedure for asymptomatic/non-COVID-19 patients and known COVID-19 positive patients

When considering the scanning of COVID-19 positive patients and those without signs or symptoms of COVID-19, careful planning is required to protect staff and patients. The rate of asymptomatic infection among healthcare workers likely reflects the general community transmission trend and there is a risk of transmission in either direction.^7^

All outpatients are routinely contacted prior to ^18^FDG-PET/CT to ask whether they have symptoms or are self-isolating. The BNMS guidance suggests that patients who have developed symptoms are asked to contact the department once they are 48 h symptom free in order to arrange their test.^3^ Patients without signs or symptoms of COVID-19 present to the radiology reception via the general entrance. Reception staff again ask whether they have symptoms of COVID-19 or have been in contact with the infection. All patients don surgical masks on entering the department and the patient waiting area allows for 2 m distancing.

In our local protocol, when imaging patients without signs or symptoms of COVID-19, the attending staff wear standard personal protective equipment (PPE) which includes a disposable fluid resistant (type IIR) surgical mask, reusable water-resistant goggles, disposable plastic gloves and a disposable plastic apron as per Public Health England guidance.^8^ ThePET scanner, uptake room and patient toilet are cleaned between patients and at the end of the list using NHS-approved “Clinell“ GAMA Healthcare Ltd surface disinfection wipes.

Symptomatic or COVID-19 positive studies are performed in cohorts at the end of the list with at least 1 h between the last non-COVID booking and the symptomatic booking to ensure no other patients are present in the department.

COVID-19 positive outpatients access the department under the direction of an escort in standard PPE via a separate entrance than that used by the general public.All patients don surgical masks on entering the department and attending staff wear standard PPE. The PET/CT scanner, uptake room and patient toilet are cleaned between positive patients and at the end of the list.^6,9^

### Case review of imaging findings in COVID-19 on ^18^FDG-PET/CT

Four patients who were RT-PCR proven COVID-19 were imaged with ^18^FDG-PET/CT between 14 and 22 days of testing positive. Two asymptomatic patients undergoing routine ^18^FDG PET/CT had radiological findings indeterminate for COVID-19 and were retrospectively analysed. All patients consented to the use of their anonymised images for training and research purposes.

The patients underwent a single-injection/dual-imaging protocol with ^18^F-FDG using a standard local procedure. Patient blood glucose levels were < 10  mmol l^−1^. A dose of 4 MBq/kg (up to a maximum of 400 MBq) ^18^F-FDG was injected intravenously and scanning commenced 60 ± 10 min after injection. Hybrid PET/CT scans were acquired by a Discovery 710 system (General Electric (GE), Chicago, IL). The patients were scanned in supine position with 2.5 min per bed position. A standard CT scan was acquired with 120-kV tube voltage.

All ^18^FDG-PET/CT scans were independently assessed by two experienced nuclear medicine radiology consultants using Q.Clear reconstructions (GE, Chicago, IL). A summary of patient demographics, clinical characteristics and imaging findings can be found in Table 1.

**Table 1.** Patient demographics, clinical characteristics and imaging findings

Patient number	1	2	3	4	5	6
**Pattern of parenchymal change**	Classic	Classic pattern, atypical location	Indeterminate	Indeterminate	Alternative diagnosis more likely	Normal
**RT-PCR status**	Positive	Positive	Not tested	Not tested	Positive	Positive
**Sex (M/F**)	F	M	M	F	F	M
**Age (years**)	52	60	71	67	60	39
**Febrile (Y/N**)	Y	Y	N	N	Y	Y
**Respiratory symptoms**	N	Pleuritic chest pain, tachypnoea, hypoxia	N	N	N	N
**Comorbidities**	AML	Follicular NHL	DLBC lymphoma	Myeloma	No	Histiocytic sarcoma
**Parenchymal change on CXR**	No	Yes	-	-	Yes	No
**CRP (mg/l**)	284	13	17	-	346	72
WBC (10*9/l)	Low (0.4)	Low (2.8)	Low (3)	Normal (5.2)	Low (0.3)	High (75.2)
Lymphocytes (10*9/l)	Low (0.2)	Low (0.6)	Low (1.1)	Low (0.8)	Low (0.3)	Normal (3.8)
**Procalcitonin (μg/L**)	0.19	0.17	-	-	0.27	0.4
**Interval between RT-PCR positive result and PET (days**)	18	14	-	-	22	15
**Reconstruction algorithm**	Q Clear	Q Clear	Q Clear	Q Clear	Q Clear	Q Clear
**No. affected lobes**	4	3	2	3	3	0
**Low dose CT features (GGO or consolidation**)	GGO &consolidation	GGO &consolidation	GGO	GGO	GGO & consolidation	Not detected
**SUV max parenchymal change**	8.1	8	2.3 LUL	3.1 LUL	12	
**LN (Y/N**)	Yes	Yes	No	No	Yes	No
**LN SUVmax**	3.8	8			4.4	
**Other findings**	Multifocal mild tracer uptake in liver and spleen, improved from previous.	Large, chronic pleural effusions. NHL mediastinal lymphadenopathy pre-dates Covid-19 infection.	Pulmonary fibrosis. Residual uptake in mesenteric node.	Numerous lytic bone lesions	Patchy colitis affecting entire colon	Mildly avid pelvic/iliac/inguinal LN's. Disease within central and proximal appendicular skeleton

CRP, C-reactive protein; F, female; GGO, ground glass opacities; LN, lymph node; M, male; No, number; SUVmax, maximum standardized uptake value; WBC, white blood cells.

### Patient 1: classic pattern of COVID-19

**Figure 1.**
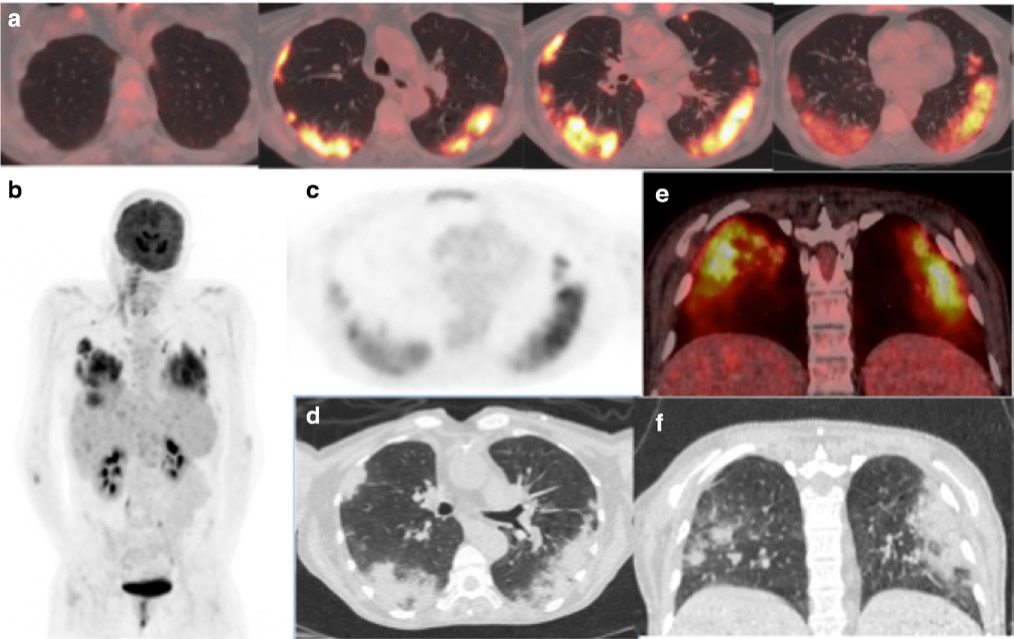
(a) Axial-fused thoracic ^18^FDG-PET/CT, (b) maximum-intensity projection, (c) ^18^FDG-PET thorax, (d) axial low-dose CT thorax (e) coronal fused ^18^FDG-PET/CT, (f) coronal thoracic CT. The images demonstrate hypermetabolic patchy peripheral consolidation and GGO in four lobes and subcarinal lymph node. ^18^FDG-PET, 18-fludeoxyglucose-positron emmision tomography; GGO, ground glass opacity.

A 52-year-old female with known transformation of myelodysplastic syndrome to acute myeloid leukaemia presented neutropaenic. 5 months earlier, she had developed numerous low attenuation FDG avid areas throughout the liver and spleen that persisted on interval ^18^FDG-PET/CT, thought potentially leukaemic or infective in nature. Liver biopsy demonstrated extensive steatosis only. A follow-up^18^FDG-PET/CT was requested to assess the response to antibiotic treatment (Figure 1). 18 days prior to the described ^18^FDG-PET/CT, the patient reported a low-grade fever, was lymphopaenic with an elevated CRP and procalcitonin of 0.19 µg l^−1^ (<0.05 µg l^−1^). RT-PCR was positive for COVID-19. A chest radiograph performed at the time was normal.

The low dose CT component of the ^18^FDG-PET/CT scan demonstrated bilateral, peripheral mid- and lower-zone predominant patchy consolidation with adjacent ground glass opacities (GGOs) in the pattern of an organising pneumonia. There was moderately increased uptake in the parenchymal change (SUVmax 8.1). A morphologically normal subcarinal lymph node was mildly avid (SUVmax 3.8). The lung parenchymal change was typical for COVID-19pneumonia.^4,9,10^ The previously seen florid liver and spleen abnormalities had significantly improved suggesting multiple treated abscesses.

### Patient 2: classic pattern of COVID-19 in an atypical location

**Figure 2.**
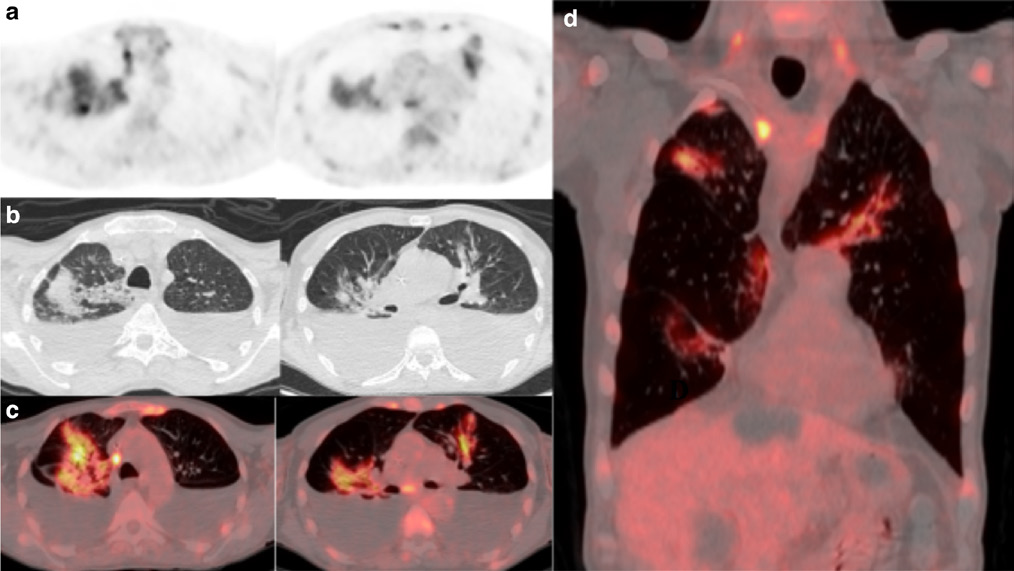
(a) ^18^FDG-PET thorax, (b) axial low-dose CT thorax, (c) axial fused thoracic ^18^FDG-PET/CT, (d) coronal fused thoracic ^18^FDG-PET/CT. The images demonstrate hypermetabolic patchy perihilar consolidation and GGO in three lobes and avid mediastinal lymph nodes. ^18^FDG-PET, 18-fludeoxyglucose-positron emmision tomography; GGO, ground glass opacity.

A 60-year-old man with relapsed follicular non-Hodgkins lymphoma presented during his second cycle of chemotherapy with pleuritic chest pain and tachypnea. Chest radiograph demonstrated longstanding large bilateral pleural effusions. The following week, he was found to be positive for COVID-19. 14 days after the RT-PCR positive test, he underwent ^18^FDG-PET/CT as an outpatient to assess disease response to chemotherapy. The ^18^FDG-PET/CT demonstrated new, moderately avid (SUVmax 8) consolidation with confluent GGO bilaterally in a mid- and upper-zone perihilar distribution (Figure 2). Large non-avid pleural effusions were present causing compressive atelectasis within the adjacent lower lobes. Pericardial, mediastinal and supraclavicular nodal avidity noted previously had reduced. The lung parenchymal change was compatible with COVID-19 pneumonia, the non-classic distribution accounted for by longstanding large bilateral pleural effusions.

### Patient 3: indeterminate pattern of COVID-19

**Figure 3.**
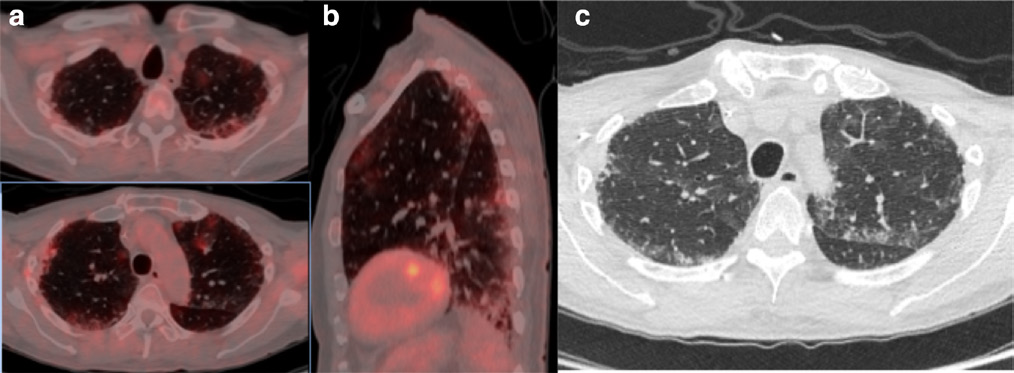
(a) Axial fused thoracic ^18^FDG-PET/CT, (b) sagittal fused thoracic ^18^FDG-PET/CT, (c) axial low-dose CT thorax. The images demonstratemultifocal biapical mildly avid GGO. ^18^FDG-PET, 18-fludeoxyglucose-positron emmision tomography; GGO, ground glass opacity.

An asymptomatic 71-year-old man with diffuse large B-cell lymphoma underwent ^18^FDG-PET/CT to assess treatment response to chemotherapy (Figure 3). A treatment response was demonstrated with residual focal uptake in the left mesentery (Deauville 4). Thoracic CT demonstrated new multifocal parenchymal GGO in the lung apices in a peripheral and peribronchovascular distribution associated with mild tracer uptake (SUVmax 2.3). The changes were non-specific and indeterminate for COVID-19 pneumonia. The patient was advised to commence self-isolation and being asymptomatic; the patient did not undergo RT-PCR testing.

### Patient 4: indeterminate pattern of COVID-19

**Figure 4.**
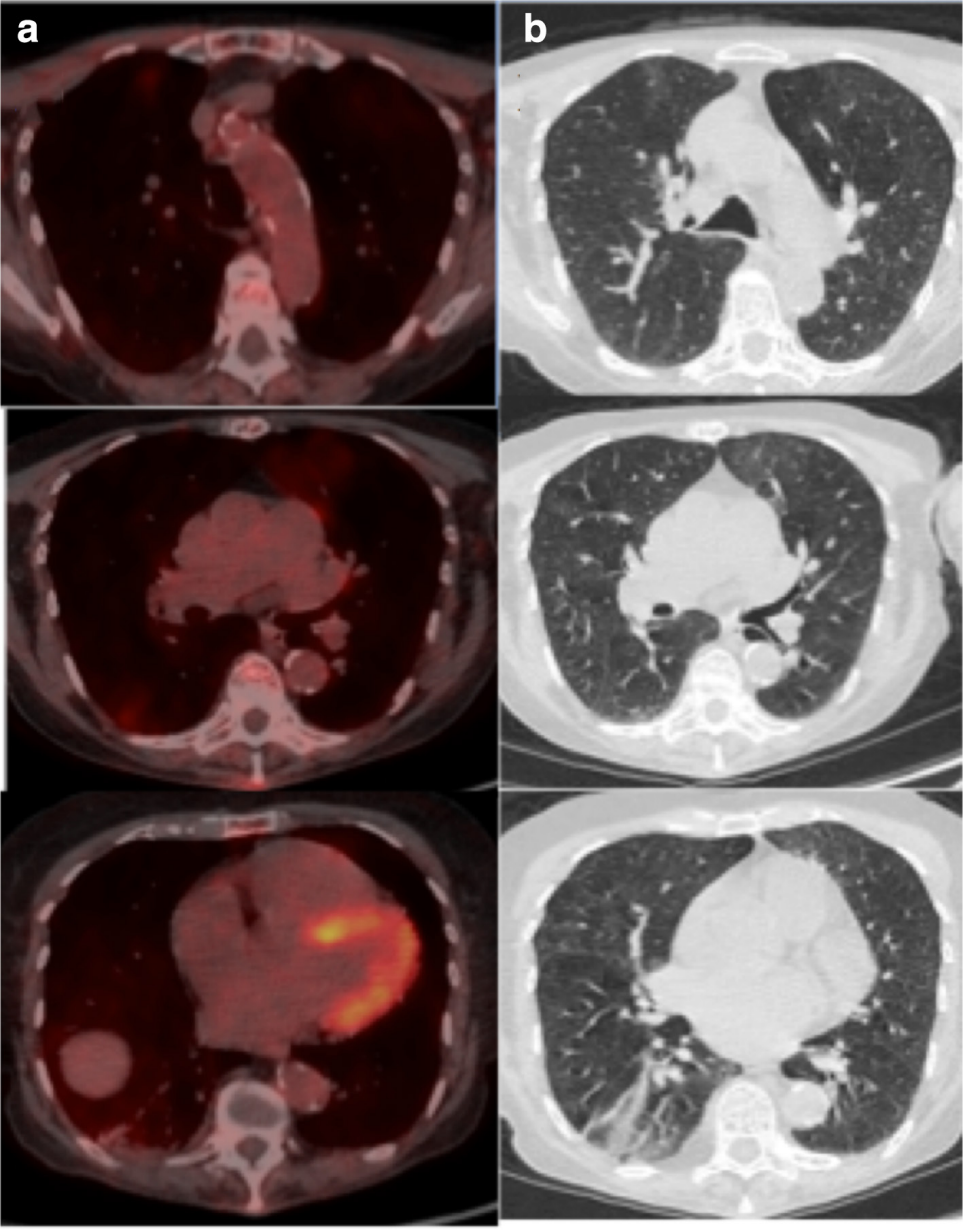
(a) Axial fused thoracic ^18^FDG-PET/CT, (b) axial low-dose CT thorax. The images demonstrate hypermetabolic GGO in three lobes with plugging and atelectasis in the lower lobes. ^18^FDG-PET, 18-fludeoxyglucose-positron emmision tomography; GGO, ground glass opacity.

A 67-year-old female with myeloma and recent hypercalcaemia was restaged with ^18^FDG-PET/CT as an outpatient (Figure 4). She had no respiratory symptoms and there was no clinical suspicion of COVID-19. The low dose CT component demonstrated patchy GGO in the upper lobes with corresponding mild avidity (SUVmax 3.1). There was longstanding volume loss and endobronchial plugging in the lower lobes. No nodal avidity. The GGO was new since the previous cross-sectional study 2 months earlier and was most likely non-COVID-19 disease but technically indeterminate for COVID-19 pneumonia. The clinical team advised the patient and co-habitants to commence self-isolation in accordance with the latest Government advice. Being asymptomatic, the patient did not undergo RT-PCR testing.

### Patient 5: pattern of alternative diagnosis in known COVID-19

**Figure 5.**
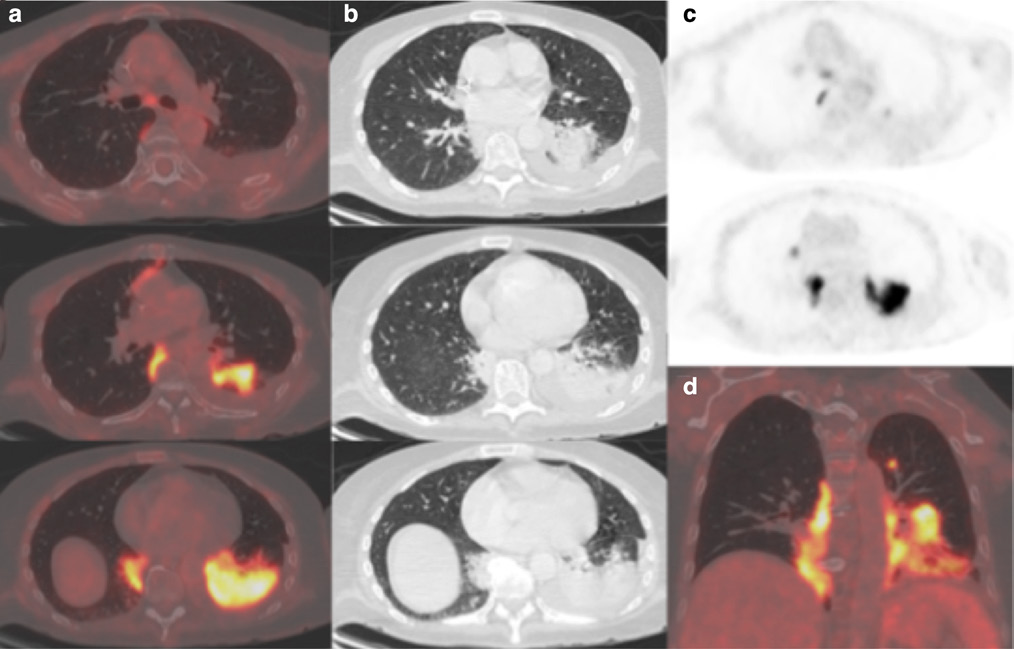
(a) Axial fused thoracic ^18^FDG-PET/CT, (b) axial low-dose CT thorax, (c) ^18^FDG-PET thorax, (d) coronal fused ^18^FDG-PET/CT. The images demonstrate hypermetabolic patchy consolidation in the lower lobes and left upper lobe. Mildly avid subcarinal lymph node. ^18^FDG-PET, 18-fludeoxyglucose-positron emmision tomography.

A 60-year-old female with aplastic anaemia was admitted with fevers and diagnosed with neutropenic sepsis. Work-up included two CT thorax/abdomen/pelvis and chest radiographs, which showed evolving tree-in-bud change in the left lower lobe but no ground glass opacification. Blood cultures grew a vancomycin resistant enterococcus. Initial swabs for COVID-19 were negative; however, on Day 26, she tested positive. 22 days after testing positive, she remained septic despite an appropriate antibiotic course with a CRP of 346mg/l, neutropaenia and procalcitonin of 0.27µg l^−1^. She underwent an ^18^FDG-PET/CT to investigate an alternative source of sepsis. Imaging showed relatively dense, amorphous consolidation in the medial and posterobasal segments of the left lower lobe in a peripheral and peribronchovascular distribution (Figure 5). The left lobar consolidation was markedly avid (SUVmax 12). Further smaller moderately avid foci were present in the left upper lobe and anteromedial basal segment of the right lower lobe (SUVmax 8.5). A morphologically normal subcarinal lymph node was mildly avid (SUVmax 4.4). Other findings included a small non-avid parapneumonic pleural effusion and patchy colitis. Colitis has been described in COVID-19 positive patients.^11^ The bilateral multilobar distribution has been described in viral infection, however the more confluent lobar consolidation and effusion was more consistent with an alternative diagnosis such as superimposed bacterial pneumonia/aspiration pneumonia.

### Patient 6: normal thoracic imaging in known COVID-19

**Figure 6.**
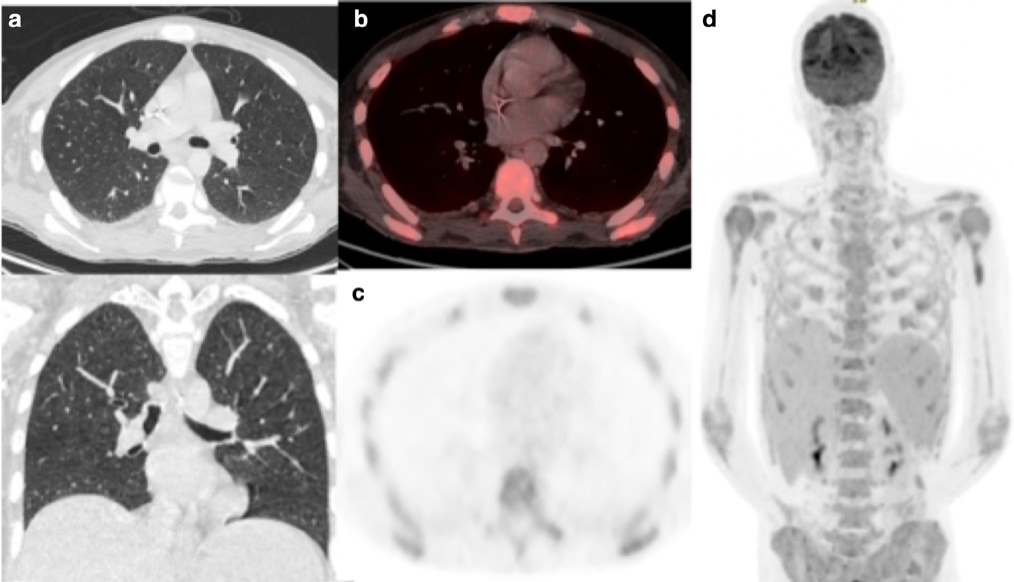
(a) Axial low-dose CT thorax, (b) axial fused thoracic ^18^FDG-PET/CT, (c) ^18^FDG-PET thorax, (d) Maximum-intensity projection. The images demonstrate diffuse marrow uptake. No lung parenchymal change or increased tracer uptake. ^18^FDG-PET, 18-fludeoxyglucose-positron emmision tomography.

A 39-year-old man with histiocytic sarcoma had been admitted following his third cycle of chemotherapy with seizures and C-difficile positive diarrhoea. RT-PCR performed 2 weeks later was positive for COVID-19. 29 days after presentation and 15 days after testing positive, he remained febrile and underwent ^18^FDG-PET/CT to guide autologous transplant. The low-dose CT component did not demonstrate parenchymal abnormality, nor was there uptake in the lungs or lymph nodes (Figure 6). Positive findings include diffuse marrow uptake with abnormal bone texture, progressive splenomegaly and pelvic lymphadenopathy felt to represent non-COVID-19 pathology.

## Discussion

Whilst a typical pattern of COVID-19 infection can be demonstrated on CT, this is not diagnostic and imaging is not recommended as a screening tool.^2,9^ Patient 1 demonstrated a typical pattern of COVID-19, described as peripheral GGO and/or nodular GGO that are bilateral, affecting two or more lobes.^4,10,12^ The presence and pattern of parenchymal change depends on when infected patients are imaged. GGO usually develops between days 0 and 4, peaking at days 6–13^4,10^. Patient 1 was imaged on Day 18 and demonstrated the most florid, typical GGO pattern within our series. Later in the course of the disease, the frequency of consolidation increases, as seen in patients 2 and 5 who were imaged at days 14 and 22 respectively. Interindividual variability is also likely.

Imaging findings on the CT component can range from diffuse GGO, linear, curvilinear or perilobular opacities to bronchocentric and peripheral consolidation and depend on the time course of infection.^10,12^ Findings may mimic alternative pathology and concomitant processes may be present.^10^ For example, the CT component of Patient 5 demonstrates bacterial or aspiration pneumonia in someone who had a recent RT-PCR swab positive for COVID-19. Whilst some thoracic findings persist beyond 28 days, many resolve. Patient 6 tested positive 15 days before the ^18^FDG-PET/CT and demonstrated no discernible lung parenchymal abnormality, highlighting the heterogeneity of this disease.

Possible thoracic findings are summarised in Table 2, modified from the British Society of Thoracic Imaging (BSTI) decision tool.^12^

**Table 2.** Potential COVID-19 patterns seen on CT component of ^18^FDG-PET/CT

Pattern	Description
**Typical COVID-19**	Mid and lower zone predominant, peripheral predominant GGO Multifocal consolidation ≥2 lobes Crazy paving Reverse halo/ Attoll sign
**Probable COVID-19**	Mid and lower zone predominant bronchocentric consolidation Minimal GGO
**Indeterminate COVID-19**	Typical or probable pattern but with unilateral or upper zone predominance Does not fit non-Covid-19 pattern
**Alternative pathology to COVID-19**	Cavitation Unifocal lobar pneumonia Effusions Tree-in-bud pattern Mass-like consolidation

^18^FDG-PET, 18-fludeoxyglucose-positron emmision tomography; GGO, ground glass opacity..

The added value of ^18^FDG-PET/CT as a functional imaging modality relies on its ability to identify metabolically active disease in the early stages, predating structural change appreciated on conventional techniques. By detecting the active phase of an infectious or inflammatory condition, it may be theoretically possible to diagnose and monitor disease progression.^13^ Deng and colleagues support the possible utility of ^18^FDG-PET/CT as a sensitive tool to detect and monitor inflammatory diseases such as viral pneumonia, although no feasibility study currently exists in COVID-19.^14^ We do not advocate this as part of current clinical practice and further research would be needed before this is considered.

It must be remembered that the cohort of patients predominantly imaged with ^18^FDG-PET/CT are cancer patients and/or immunosuppressed, therefore particularly vulnerable to COVID-19. Joob highlights that ^18^F-FDG PET/CT is not recommended in infectious pneumonia, warning of the risk of spreading COVID-19 in nuclear medicine departments given the long procedure period compared with conventional CT.^15^ We feel that we have mitigated these risks by delaying 1–2 weeks for urgent cases and 4–6 weeks for non-urgent cases, cohorting the patients at the end of the day and cleaning equipment as per GE and infection control guidance.

Albano and colleagues found COVID-19 parenchymal change in asymptomatic patients undergoing ^18^FDG-PET/CT in 9% of cases, based on retrospective review of 65 asymptomatic patients.^16^ Since GGO is the earliest reported finding, it is possible that asymptomatic patients 3 and 4 who had multilobar hypermetabolic GGO were imaged during pre-clinical manifestation of the infection. Subsequently, the clinical team was contacted to ensure that the patient and co-habitants commence self-isolation in accordance with the latest Government advice. The ^18^FDG-PET/CT reporting radiologist can add further value by identifying incidental subclinical infection and recommending immediate self-isolation.

Whilst not advocated by BNMS or European guidance, it may be beneficial to review localising CT images of asymptomatic patients that include the chest for the presence of COVID-19 as soon as possible and/or before the next patient enters the imaging suite. This is logistically difficult to achieve and, on the advice of our infectious disease department, we wear standard PPE for all patients and clean the uptake rooms and scanner between every patient. We suggest a local view on this is formed, taking into account local prevalence of COVID and supplies of PPE. A standardised reporting template has been proposed by the BSTI for the reporting of chest radiographs and thoracic CT. This includes categorising findings as Normal, Classic/Probable COVID-19, Indeterminate for COVID-19 and Non-COVID-19. They also propose stating the disease distribution and extent.^12^ This could be adopted by nuclear medicine reporters to both highlight potential COVID-19 infection to the clinical team and facilitate future research.

Benign GGOs demonstrate significantly higher ^18^FDG uptake on PET/CT than malignant GGOs/GGNs.^14,17^ Similarly, earlier case series’ by Qin et al and Setti et al suggest that COVID-19 pulmonary abnormalities display moderate to high ^18^FDG uptake (GGO SUVmax range 4.6–12.2 and GGO SUVmax range 4.3–11.3 respectively).^18,19^ Correspondingly, our cohort demonstrate moderate-to-marked GGO uptake (SUVmax 8–12).

Two cases had ^18^FDG uptake in morphologically normal mediastinal lymph nodes. Nodes were mildly avid (SUVmax range 3.8–4.4), as demonstrated in earlier case series.^14,16,18,19^ This reflects an infective/inflammatory aetiology and is not necessarily specific to COVID-19 as previously postulated.^18^ Lymphadenopathy was not described and is rarely reported on thoracic CT.^10^

A limitation of ^18^FDG-PET/CT when assessing lungs is that routinely the CT component is low dose (120-kV tube voltage) and not performed on a breath-hold. Reducing the tube voltage improves image contrast to aid accurate localisation but in turn can mask GGO. Conversely, motion artefact generated on a free-breathing CT thorax can artificially create GGO, particularly in the lower lobes. Overall ^18^FDG-PET/CT has a lower detection rate for smaller GGO and exhibits no clear advantage for the detection of pure GGO that are not metabolically active.^17^ In inflammatory aetiologies such as active COVID-19, we can expect elevated glucose metabolism, however the sensitivity of ^18^FDG-PET/CT in detecting post-infective GGO that is no longer hypermetabolic is potentially limited.

## Learning points

- ^18^FDG PET/CT can be performed in COVID-19 positive patients if clinically indicated with precautions to protect staff and patients.
- COVID-19 positive patients may have a spectrum of findings on ^18^FDG-PET/CT from a normal study to the classically described moderately FDG avid, peripheral GGOs in at least two lobes.
- A normal^18^FDG-PET/CT does not exclude COVID-19 and clinicians should remain suspicious if symptoms and biochemical profiling are suggestive.
- Radiologists and nuclear medicine physicians should be aware of the thoracic appearances that can be seen in unsuspected COVID-19 infection in routine outpatient ^18^FDG-PET/CT’s in order to flag to referring clinicians when necessary.
